# Soluble ST2 and Interleukin-33 Levels in Coronary Artery Disease: Relation to Disease Activity and Adverse Outcome

**DOI:** 10.1371/journal.pone.0095055

**Published:** 2014-04-21

**Authors:** Svitlana Demyanets, Walter S. Speidl, Ioannis Tentzeris, Rudolf Jarai, Katharina M. Katsaros, Serdar Farhan, Konstantin A. Krychtiuk, Anna Wonnerth, Thomas W. Weiss, Kurt Huber, Johann Wojta

**Affiliations:** 1 Department of Internal Medicine II, Medical University of Vienna, Vienna, Austria; 2 Department of Laboratory Medicine, Medical University of Vienna, Vienna, Austria; 3 3rd Medical Department for Cardiology and Emergency Medicine, Wilhelminenhospital, Vienna, Austria; 4 Core Facilities, Medical University of Vienna, Vienna, Austria; 5 Ludwig Boltzmann Cluster for Cardiovascular Research, Vienna, Austria; University Hospital Medical Centre, Germany

## Abstract

**Objectives:**

ST2 is a receptor for interleukin (IL)-33. We investigated an association of soluble ST2 (sST2) and IL-33 serum levels with different clinical stages of coronary artery disease. We assessed the predictive value of sST2 and IL-33 in patients with stable angina, non-ST elevation myocardial infarction (NSTEMI) and ST elevation myocardial infarction (STEMI).

**Methods:**

We included 373 patients of whom 178 had stable angina, 97 had NSTEMI, and 98 had STEMI. Patients were followed for a mean of 43 months. The control group consisted of 65 individuals without significant stenosis on coronary angiography. Serum levels of sST2 and IL-33 were measured by ELISAs.

**Results:**

sST2 levels were significantly increased in patients with STEMI as compared to patients with NSTEMI and stable angina as well as with controls. IL-33 levels did not differ between the four groups. During follow-up, 37 (10%) patients died and the combined endpoint (all cause death, MI and rehospitalisation for cardiac causes) occurred in 66 (17.6%) patients. sST2 serum levels significantly predicted mortality in the total cohort. When patients were stratified according to their clinical presentation, the highest quintile of sST2 significantly predicted mortality in patients with STEMI, but not with NSTEMI or stable coronary artery disease. sST2 was a significant predictor for the combined endpoint in STEMI patients and in patients with stable angina. Serum levels of IL-33 were not associated with clinical outcome in the total cohort, but the highest quintile of IL-33 predicted mortality in patients with STEMI.

**Conclusions:**

Serum levels of sST2 are increased in patients with acute coronary syndromes as compared to levels in patients with stable coronary artery disease and in individuals without coronary artery disease. sST2 and IL-33 were associated with mortality in patients with STEMI but not in patients with NSTEMI or stable angina.

## Introduction

ST2 (also known as T1 or interleukin (IL)-1 receptor-like-1 (IL1RL1)) is a member of the Toll-like/IL-1-receptor superfamily [1], [2]. ST2 was known for more than 15 years as an orphan receptor before its ligand cytokine IL-33 was discovered in 2005 [3]. The soluble ST2 (sST2) and the transmembrane ST2 (ST2L) isoforms arise from a dual promoter system to drive differential mRNA expression [4]. ST2 is established as a selective marker of T helper type 2 (Th2) lymphocytes [1]. In addition, ST2 is also expressed on mast cells, epithelial, endothelial and smooth muscle cells, rat neonatal cardiac fibroblasts and cardiac myocytes [2], [5]–[8]. IL-33 is a dual-function cytokine, which is either expressed in the nucleus or activates cells via the receptor complex consisting of ST2L and IL-1 receptor accessory protein (IL-1RaP) [2], [3]. IL-33 is a multifunctional immunomodulatory cytokine that acts both pro- and anti-inflammatory depending on the disease state or experimental conditions [9], [10]. sST2 acts as a decoy receptor by binding free IL-33 and preventing its signaling through ST2L [7].

Weinberg et al. were the first who showed increased sST2 levels in patients after myocardial infarction, which correlated positively with peak creatine kinase and negatively with left ventricle ejection fraction [6]. The same group revealed a predictive value of sST2 in patients with ST-elevation myocardial infarction (STEMI) by showing that baseline levels of sST2 were significantly higher in those patients who died or developed new congestive heart failure during short-term follow-up (30 days) [11]. Multi-marker approach revealed that the combination of sST2 and N-terminal prohormone B-type natriuretic peptide (NT-proBNP) significantly improves risk stratification in STEMI patients during 30-day follow-up [12]. A possible pathophysiological importance of sST2 in infarct remodeling was further confirmed by a study of Weir et al. who found relationships between sST2 and infarct magnitude/evolution over 24 weeks of observation in patients with acute myocardial infarction with resultant left ventricle systolic dysfunction [13].

In non-ST elevation myocardial infarction (NSTEMI), sST2 was related to 1-year mortality, to cardiovascular death/heart failure at 30 days and 1 year as well as to death, heart failure readmission, and reinfarction during the long term, respectively [14]–[16]. Until now there is no data on the predictive value of sST2 in stable coronary artery disease (CAD).

Thus, increased levels of the soluble receptor for IL-33, sST2, are recognized as a marker of poor prognosis in patients with myocardial infarction and heart failure [9], [11], [17]. However, the prognostic value of circulating IL-33 in cardiovascular disease is not clear. Recently, Dhillon et al. showed that elevated sST2 and IL-33 were both associated with increased mortality in STEMI patients [18], but in patients with NSTEMI only sST2 and not IL-33 levels were related to adverse events such as all-cause mortality, heart failure hospitalization, and reinfarction [16]. Our group found recently that an increase of IL-33 serum levels after coronary stent implantation is associated with coronary in-stent restenosis in patients with both stable and unstable CAD [19].

Although increased levels of sST2 in patients with STEMI and NSTEMI were shown over different periods of time [6], [11], [13], [14], a comparison of sST2 and IL-33 concentrations in patients with stable angina and acute coronary syndromes and individuals without CAD was not studied before and was the aim of our study. Additionally, we determined the long-term predictive value of sST2 and IL-33 levels in the examined groups of patients. It should be emphasized, however, that it was not the main objective of this work to identify yet another clinical biomarker or point of care test for the diagnosis of acute coronary syndrome or for the clinical risk prediction in stable coronary artery disease. Rather it was the aim of this study to gain additional insights in the pathophysiological role of the ST2/IL-33 system in atherosclerotic disease and myocardial infarction.

## Methods

### Ethics Statement

The study protocol conforms to the ethical guidelines of the Declaration of Helsinki as reflected in a priori approval by the ethics committee of the city of Vienna, Austria. All subjects provided written consent.

### Study Population

Blood samples were taken from 373 consecutive patients with angiographically proven CAD. From these patients 178 had stable angina (SA), 97 NSTEMI, and 98 STEMI, respectively. In addition we included 65 subjects without evidence of coronary stenosis on coronary angiogram. Exclusion criteria were presence of autoimmune diseases, chronic infections, hepatic or renal disorders. Aspirin and unfractionated heparin were administered per standard practice. Clopidogrel therapy was started either on the day before angiography or immediately after stent implantation with 300 mg. Other medications such as beta-blockers and angiotensin-converting-enzyme inhibitors were given as appropriate. Statin therapy was routinely administered to all patients according to international guidelines. At inclusion time new antiplatelet drugs like ticagrelor and prasugrel were not yet available.

### Blood Samples

Blood samples were taken under fasting conditions directly before coronary angiography. Venous blood was drawn from the antecubital vein with minimal tourniquet pressure into serum separator tubes. Samples were allowed to clot for 30 minutes (min) before centrifugation (4°C; 3,000 g for 15 min) and stored at −80°C until use.

### Laboratory Measurements

sST2 and IL-33 were measured with specific enzyme-linked immunosorbent assays (ELISAs; both from R&D Systems, Minneapolis, MN, USA). Minimum detection limit for sST2 was 31.3 pg/mL; for IL-33 minimum detection limit was 23.4 pg/mL. Laboratory determinations were performed by investigators that were blinded to clinical characteristics and patients’ outcome.

### Follow-up

Patients were followed for a mean of 43 months for the occurrence of death and a combined clinical endpoint (all cause death, myocardial infarction and rehospitalisation for cardiac causes).

### Statistical Analysis

We estimated a mortality of 10% in this cohort of patients with STEMI, NSTEMI and SA. Therefore, calculation of sample size revealed that we would need at least 320 patients to detect a difference in sST2 serum levels of 50% with a power of 80% and significance level (two-tailed) of 0.05 [20]. Variables were tested for normal distribution using the Kolmogorov-Smirnov test. Continuous variables were expressed as mean ± SD or as median, interquartile range according their distribution. Categorical variables were summarized as counts and percentages and were compared by the chi-square or by Fisher exact test as appropriate. Continuous variables were compared using Student’s t-test when normally distributed and by Mann-Whitney-U test when not normally distributed. ANOVA followed by Bonferroni-Holm multiple comparisons correction was carried out in case when more than 2 groups were compared. Spearman correlation was used to determine the correlation between level of sST2 and cardiovascular risk factors or level of IL-33 and cardiovascular risk factors. Multivariate analysis was performed with the Cox proportional hazard model in which mortality was used as dependent variable and potentially confounding baseline variables were used as independent variables. Baseline variables were selected for the model if they appeared to be imbalanced between patients with and without survival indicated by a univariate p-value <0.20 or if they were correlated with ST2 or IL-33, respectively. A value of p<0.05 (two-tailed) was considered statistically significant. All statistical analyses were performed with the statistical software package SPSS version 18.0 (SPSS, Inc., Chicago, Illinois).

## Results

### Study Population Characteristics

Demographic data of the entire cohort, controls and patients with SA, NSTEMI and STEMI, respectively are shown in the Table 1. There were no differences in regard to gender, age, diabetes, and kidney function between the four study groups. Although controls had a higher body mass index (BMI), they were less likely to have hyperlipidemia as compared to patients with CAD. Patients with STEMI were more likely to be smokers (p = 0.02) but less likely to be hypertensive (p = 0.001) (Table 1).

**Table 1:** Baseline characteristics of study population.

	Total (n = 438)	Controls (n = 65)	SA (n = 178)	NSTEMI (n = 97)	STEMI (n = 98)	P-value
Age (yrs)	64.4±12.1	62.6±8.9	65.9±11.5	64.6±13.5	62.9±13.7	0.14
Male sex, N (%)	279 (63.7)	32 (49.2)	122 (68.5)	60 (61.9)	65 (66.3)	0.06
Hypertension, N (%)	328 (74.9)	46 (70.8)	142 (79.8)	80 (82.5)	60 (61.2)	0.001
Smoker, N (%)	130 (29.7)	20 (30.8)	43 (24.2)	26 (26.8)	41 (41.8)	0.02
Hyperlipidaemia, N (%)	324 (74.0)	35 (53.8)	140 (78.7)	76 (78.4)	73 (74.5)	0.001
Diabetes, N (%)	95 (21.7)	18 (27.7)	39 (21.9)	15 (15.5)	23 (23.5)	0.29
BMI (kg/m^2^)	27.9±4.6	28.7±6.0	27.4±4.1	27.9±4.2	27.6±4.6	0.005
Serum creatinine (mg/dL)	1.04±0.64	1.02±0.28	1.08±0.93	1.01±0.31	1.03±0.33	0.80

SA denotes stable angina, NSTEMI denotes non-ST-elevation myocardial infarction, STEMI denotes ST-elevation myocardial infarction, CAD denotes coronary artery disease, BMI denotes body mass index.

### Serum Levels of sST2 and IL-33 in Relation to Cardiovascular Risk Factors

IL-33 was detectable in 46.1% of study participants and was below detection limit in 53.9%. IL-33 levels were significantly higher in females (p<0.05) and in non-smokers (p<0.05). Serum levels of sST2 and IL-33 showed a significant correlation (r = 0.38, p<0.000001). sST2 levels correlated positively with creatinine levels (r = 0.126; p = 0.009) but were not associated with classic cardiovascular risk factors.

### sST2 and IL-33 Concentrations in Persons without CAD and Patients with Different Clinical Stages of CAD

**Figure 1:**
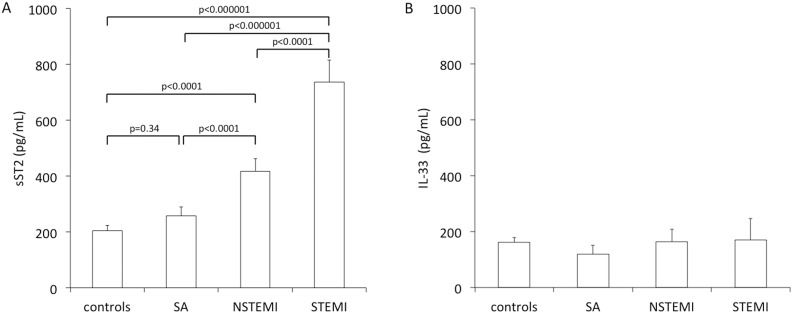
sST2 and IL-33 serum levels in controls and according clinical presentation of coronary artery disease. sST2 (A) and IL-33 (B) serum levels were measured as described under “Methods”. SA denotes stable angina, NSTEMI denotes non-ST-elevation myocardial infarction, STEMI denotes ST-elevation myocardial infarction. Mean ± standard error of mean.

Study participants showed significantly different sST2 serum levels according to their clinical presentation (Figure 1A). Controls (163, IQR 114–260 pg/mL) showed similar sST2 levels as compared to patients with SA (169, IQR 79–260 pg/mL; p = 0.34). Patients with STEMI showed the highest serum levels of sST2 (453, IQR 313–688 pg/mL) as compared to patients with NSTEMI (269, IQR 157–496 pg/mL; p<0.0001), patients with SA (p<0.000001) and controls (p<0.000001). In addition, patients with NSTEMI showed significantly higher levels of sST2 as compared to patients with stable CAD (p<0.0001) and controls (p<0.0001, Figure 1A). In contrast, IL-33 was detectable in 58.5%, 42.4%, 46.8% and 43.6% of individuals without CAD, patients with SA, NSTEMI and STEMI, respectively (p = NS) and IL-33 serum levels did not differ between the respective groups (p = NS, Figure 1B).

### Relation of sST2 and IL-33 Serum Levels to Outcome

**Figure 2:**
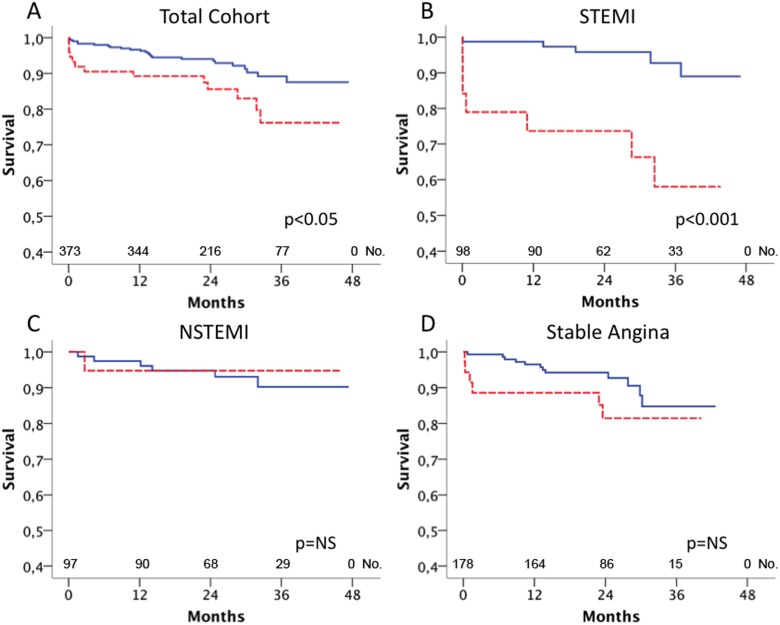
Relation of sST2 serum levels to mortality. sST2 serum levels were measured as described under “Methods”. Kaplan Meier survival curves for the highest quintile of sST2 serum levels (red, dashed line) vs. the lower four quintiles of sST2 (blue, full line) in all patients (A), patients with ST-elevation myocardial infarction (B), non-ST-elevation myocardial infarction (C) and stable angina (D).

**Figure 3:**
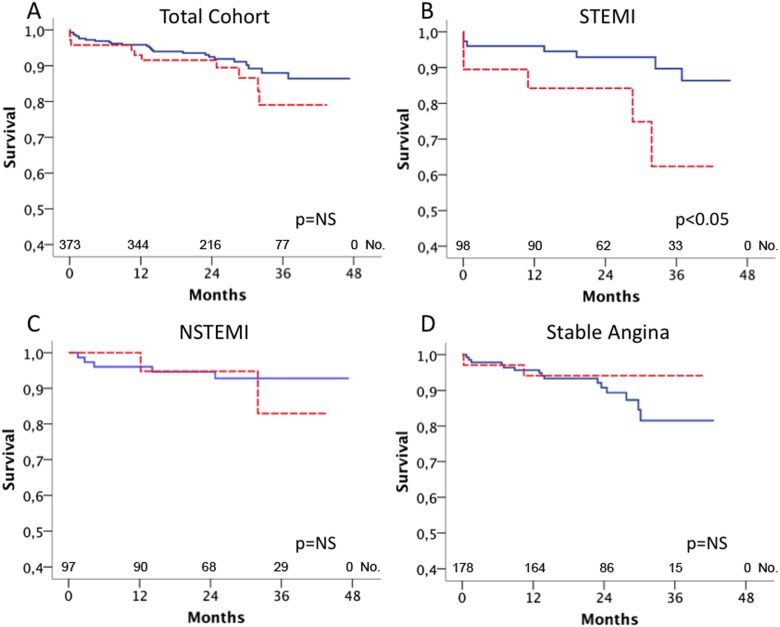
Relation of IL-33 serum levels to mortality. IL-33 serum levels were measured as described under “Methods”. Kaplan Meier survival curves for the highest quintile of IL-33 serum levels (red, dashed line) vs. the lower four quintiles of IL-33 (blue, full line) in all patients (A), patients with ST-elevation myocardial infarction (B), non-ST-elevation myocardial infarction (C) and stable angina (D).

Mean follow-up time was 43 months. During follow-up, 37 (10%) patients died and the combined endpoint occurred in 66 (17.6%) patients. Patients who died during follow-up showed significantly higher serum levels of sST2 as compared to survivors (415, IQR 136–1158 pg/mL vs. 241, IQR 129–426 pg/mL; p<0.05). The highest quintile of sST2 serum levels significantly predicted mortality in the total cohort (p<0.05, Figure 2A). When the total cohort was stratified according clinical presentation, sST2 was a strong predictor of mortality in patients with STEMI (p<0.001, Figure 2B) but not in patients with NSTEMI (p = NS, Figure 2C) or SA (p = NS, Figure 2D), respectively. sST2 serum levels significantly predicted the occurrence of the combined endpoint in patients with STEMI (p<0.005) and patients with SA (p<0.05), but not in patients with NSTEMI (p = NS, data not shown). The rate of IL-33 above detection limit was 44.1% in survivors vs. 41.7% in non-survivors (p = NS), and IL-33 serum levels were not associated with mortality in the total cohort of patients with CAD (p = NS, Figure 3A). However, when patients were stratified for their clinical presentation, the highest quintile of IL-33 was a significant predictor of mortality in patients with STEMI (p<0.05, Figure 3B) but not in patients with NSTEMI (p = NS, Figure 3C) and SA (p = NS, Figure 3D). Serum levels of IL-33 did not predict the combined endpoint in the total cohort (p = NS) and in the subgroups with SA (p = NS), NSTEMI (p = NS) and STEMI (p = NS, data not shown), respectively.

### Multivariate Analysis

Patients in the highest quintile of sST2 serum levels had a 2.1-fold risk for mortality as compared to patients in the lower quintiles (p<0.05). Multivariate analysis revealed that the fifth quintile of sST2 serum levels was a significant predictor of mortality after adjustment for clinical risk factors including creatinine levels (Table 2).

**Table 2:** Cox proportional hazard model assessing the risk for mortality according to sST2 levels.

	Odds ratio	95% confidence interval	P-value
univariate			
Patients with sST2 serum levels in quintile 1–4 (5–538 pg/mL)	1		
Patients with sST2 serum levels in quintile 5 (539–3618 pg/mL)	2.1	1.1–4.2	0.029
adjusted for age, gender, hyperlipidemia, hypertension, smoking, BMI, serum creatinine			
Patients with sST2 serum levels in quintile 1–4 (5–538 pg/mL)	1		
Patients with sST2 serum levels in quintile 5 (539–3618 pg/mL)	2.2	1.1–4.4	0.03

sST2 denotes soluble ST2, CAD denotes coronary artery disease, BMI denotes body mass index.

## Discussion

In the present study we found that sST2 levels are significantly increased in patients with acute coronary syndrome as compared to patients with stable coronary artery disease and as a control in individuals without significant stenosis on coronary angiography. Moreover we could demonstrate that patients with STEMI show higher levels of sST2 than patients with NSTEMI. However, sST2 levels were not significantly different between the control group and patients with stable angina. In addition, sST2 significantly predicted death in the total cohort over 43 months, which is mainly driven by the STEMI group. sST2 also significantly predicts the combined endpoint in the STEMI group and in the SA group. sST2 levels independently predicted mortality in these patients. IL-33 levels were not different between the patients with stable coronary artery disease and acute coronary syndrome and the control group. IL-33 levels predicted mortality only in patients with STEMI. sST2 and IL-33 levels positively correlated in this study population.

A predictive value for sST2 was shown previously in patients with NSTEMI, STEMI, as well as in apparently healthy individuals [11], [12], [14], [15], [21], [22]. We confirm the data about long-term prognostic value of sST2 in acute coronary syndrome, and showed for the first time that in addition to that, sST2 levels predicted the combined endpoint also in patients with stable coronary artery disease. Combined measurements of sST2 and IL-33 were performed recently by Dhillon et al. in patients with NSTEMI and STEMI, who revealed that sST2 levels predicted adverse outcomes in both cohorts, however, IL-33 levels were associated with increased mortality at 30-days and 1-year only in STEMI patients [18], and was not different across any endpoint in the NSTEMI cohort [16]. According with the findings of Dhillon et al. [18], we demonstrated in this study that circulating IL-33 levels predict mortality only in the patients with STEMI, but not in the patients with NSTEMI or stable angina. It should be noted that this predictive value of IL-33 was observed over a period of time of more than 3.5 years in our study.

Furthermore, we showed here for the first time that the level of circulating sST2, but not that of IL-33, is associated with the stage of disease and is increased continuously from stable CAD to NSTEMI and STEMI. Previously, genetic variability of the distal promoter of the ST2 gene was shown to be associated with angiographic severity of CAD [23]. Interestingly, we found no differences in sST2 levels between patients with stable angina and individuals without significant stenosis on coronary angiography, although both STEMI and NSTEMI patients demonstrated significantly higher sST2 serum levels than the control group. Therefore, our findings highlight increased levels of circulating sST2 specifically under destabilization of coronary artery disease.

In a community-based cohort of Framingham Heart Study, values for sST2 differ between men and women, increase with age, and are associated with diabetes and hypertension [24]. However, sST2 did not correlate with classic markers linked to CAD risk, including cholesterol, blood pressure, smoking, common carotid intima-media thickness or plaque presence in the cross-sectional pSoBid study [25]. In our cohort here, sST2 levels were not associated with classic cardiovascular risk factors such as age, sex, hypertension, family history of CAD, smoking, hyperlipidaemia or diabetes but correlated positively with creatinine levels. IL-33 levels were significantly higher in females and in non-smokers.

The IL-33/ST2 system attracted special attention of cardiologists after ST2 was identified to be inducible by mechanical strain in neonatal rat cardiac myocytes in 2002 [6], and after the identification of IL-33 as a ligand for the ST2 receptor in 2005 [3]. Two main isoforms of ST2 are known – the soluble form sST2 and the transmembrane form ST2L. The sST2 isoform lacks the transmembrane and cytoplasmic domains contained within the structure of ST2L [2], [4]. Despite its similarity to the IL-1 receptor (IL-1R), ST2 does not bind IL-1α, IL-1β, or IL-1R antagonist [1]. sST2 in the extracellular environment might bind free IL-33, thereby decreasing the concentration of IL-33 that is available for ST2L binding and reducing the biological effects of IL-33 [2], [3], [7].

Data on possible cellular sources of circulating sST2 and IL-33 as well as their regulation in cardiac cells are controversial. Rat neonatal cardiac myocytes and fibroblasts were shown to increase sST2 mRNA as well as IL-33 mRNA expression upon mechanical strain [6], [7]. Recently, IL-33 was revealed to be released by murine fibroblasts undergoing mechanical strain in the absence of cellular necrosis [26]. However, release of sST2 and/or IL-33 by human cardiac cells under the same conditions was not demonstrated before.

Data published by Bartuneck et al. showed no difference in arterial and coronary sinus levels of sST2 and thus argues against myocardial production of sST2 [27]. Those authors found IL-33 protein to be localized to endothelial cells in the human coronary artery [27]. According with these findings, we recently showed that both human macrovascular (aortic and coronary artery) and heart microvascular endothelial cells secrete sST2 protein, whereas human adult cardiac myocytes, cardiac fibroblasts and vascular smooth muscle cells do not secrete detectable amounts of sST2 antigen *in vitro* [8].

The regulation of sST2 and IL-33 seems to be development-dependent and cell- and species-specific. In neonatal rat cardiac myocytes and fibroblasts, sST2 expression is increased by IL-1β, phorbol ester, uridine triphosphate and endothelin-1, but not by TNF-α, angiotensin II, hydrogen peroxide, IL-4 or lipopolysaccharide (LPS) [6], [28], [29]. However, in human endothelial cells, sST2 was shown to be upregulated by the inflammatory cytokines IL-1β, TNF-α and by phorbol ester [27]. Additionally, proinflammatory mediators IL-1α, IL-1β, and TNF-α as well as supernatants of LPS-stimulated peripheral blood mononuclear cells led to an enhanced secretion of sST2 in cultured human adult cardiac myocytes, but not in cardiac fibroblasts [30]. IL-33 expression is increased upon treatment with phorbol 12-myristate 13-acetate and angiotensin II, but not TNF-α or IL-1β in rat neonatal cardiomyocytes and cardiac fibroblasts [7], and upon treatment with TNF-α, IL-1β, and IFN-γ in human adult cardiac myocytes and fibroblasts [8].

IL-33 and ST2 were proposed to comprise a cardioprotective signaling system because IL-33 was found to antagonize cardiac hypertrophy as well as hypoxia-induced apoptosis *in vitro* and *in vivo* and was thereby shown to protect mice from experimental pressure overload and myocardial infarction [7], [31]. Exogenous IL-33 also attenuated ischaemia/reperfusion injuries in the diabetic myocardium in mice [32]. A cardioprotective role of this system was confirmed in ST2-deficient mice [7], [31]. However, in a model of autoimmune heart disease treatment with IL-33 induced eosinophilic pericarditis and cardiac dilation, whereas sST2 prevents eosinophilia and improves systolic function [33]. Therefore, caution should be taken to transfer results from animal experiments into the clinical setting.

Data about expression of the components of IL-33/ST2 system in human heart under different pathologies are still scarce. Left ventricular (LV) ST2 mRNA levels were not significantly different in patients with aortic stenosis, congestive cardiomyopathy and stable angina pectoris with normal LV function (control group), however, IL-33 mRNA levels were lower in LV biopsies from aortic stenosis patients compared to control [27]. Our group found IL-33 mRNA expression to be positively correlated with IFN-γ and TNF-α mRNA expression in human myocardial tissue from patients undergoing heart transplantation [8]. Moreover, we demonstrated that both IL-33 and ST2 protein are expressed predominantly by endothelial cells in human heart [8].

Some limitations of the present study have to be acknowledged. First, our study is of observational nature. Accordingly, our results may be explained by unmeasured confounding factors. Therefore, we tried to control for baseline imbalances by multivariate modeling. However, the possibility of residual or undetected confounding is small but cannot be ruled out completely. In addition, IL-33 was not detectable in 54% of the study participants. Similar to our findings, Dhillon et al. found that in more than half of the patients with NSTEMI and STEMI IL-33 levels were below the lower detection limit of the assay used [16], [18]. However, IL-33 in the last quintile was statistically associated with an adverse outcome in patients with STEMI. It should also be pointed out, that the study was not undertaken and thus not designed to identify yet another clinical biomarker or point of care test for the diagnosis of acute coronary syndrome or for the clinical risk prediction in stable coronary artery disease. Our study aimed to gain additional insights in a possible pathophysiological role of the ST2/IL-33 system in atherosclerotic disease and myocardial infarction.

## Conclusions

In conclusion, we identified for the first time circulating sST2 levels to be associated with disease activity in coronary artery disease, and to be increased in patients with acute coronary syndrome as compared to patients with stable CAD and persons without CAD. Both sST2 and IL-33 were associated with mortality in patients with STEMI but not in patients with NSTEMI or stable angina over a period of time of more than 3.5 years. sST2 also significantly predicts the combined endpoint in the STEMI group and in the stable angina group. Further studies on the role of IL-33/ST2 system in cardiovascular pathologies are warranted.
